# Acupuncture ameliorates breast cancer-related fatigue by regulating the gut microbiota-gut-brain axis

**DOI:** 10.3389/fendo.2022.921119

**Published:** 2022-08-24

**Authors:** Zhuan Lv, Ruidong Liu, Kaiqi Su, Yiming Gu, Lu Fang, Yongfu Fan, Jing Gao, Xiaodi Ruan, Xiaodong Feng

**Affiliations:** ^1^ Department of Rehabilitation Center, The First Affiliated Hospital of Henan University of Chinese Medicine, Zhengzhou, China; ^2^ Department of Rehabilitation Medicine, Henan University of Chinese Medicine, Zhengzhou, China; ^3^ Department of Breast surgery, The First Affiliated Hospital of Henan University of Chinese Medicine, Zhengzhou, China

**Keywords:** cancer-related fatigue (CRF), breast cancer, acupuncture treatment, gut microbiota-gut-brain axis, chemotherapy

## Abstract

Cancer-related fatigue (CRF) is the most common side effect of chemotherapy for breast cancer (BC). Acupuncture treatment has an anti-fatigue effect and can regulate gut microbiota disturbance in fatigue patients. Related studies have shown that the gut microbiota-gut-brain axis is closely related to the occurrence of CRF. In this study, we first investigated the alterations of acupuncture on fatigue-like behavior, gut microbiota, gut inflammation and neuroinflammation response, gut barriers, HPA axis, and serum metabolomics in CRF mice after BC chemotherapy. Then, the correlation analysis of gut microbiota and other indicators was discussed. Our results showed that acupuncture treatment could exert an anti-fatigue effect and ameliorate the gut barrier, gut inflammation, neuroinflammation, and dysfunction of the HPA axis in CRF mice after chemotherapy for BC. 16S rRNA sequencing showed that acupuncture treatment could enhance the abundance of *Candidatus Arthromitus*, *Lactobacillus*, and *Clostridia_UCG-014_unclassified* and decrease the abundances of *Escherichia-Shigella*, *Burkholderia-Caballeronia-Paraburkholderia*, and *Streptococcus*. Serum metabolomics analysis showed that acupuncture treatment could regulate the differential metabolites N-methylnicotinamide, beta-glycerophosphoric acid, geranyl acetoacetate, serotonin and phenylalanine, tyrosine and tryptophan biosynthesis, taurine and hypotaurine, and beta-alanine metabolic pathways. Correlation analysis indicated that there are certain correlations between gut microbiota and gut inflammation, neuroinflammation, gut barrier, HPA axis function and serum metabolites. In conclusion, our findings revealed that the anti-fatigue mechanism of acupuncture treatment may be closely related to the gut microbiota-gut-brain axis. This study also provided a new reference for basic and clinical research on CRF after breast cancer chemotherapy.

## Introduction

Cancer-related fatigue (CRF) is the most common side effect of cancer treatment. It mainly manifests as persistent fatigue and a decline in emotional and cognitive functions and cannot be relieved by rest and sleep, resulting in the decrease of body function and quality of life (1, 2). Breast cancer (BC) is the most common cancer in women worldwide, though patient survival rates continue to improve (3). Meanwhile, CRF is more common in breast cancer survivors than in survivors of other cancers. In addition, the study found that the incidence of CRF in patients receiving chemotherapy treatment is 70%-100%, and the degree of fatigue of patients is significantly worse than that of patients without chemotherapy (4, 5). Notably, researchers have shown that chemotherapy can activate the peripheral proinflammatory cytokine network and cause central nervous system inflammation by disrupting the permeability of the physiological barrier which, in turn, causes dysfunction of the hypothalamic–pituitary–adrenal (HPA) axis, ultimately leading to CRF (6).

The gut microbiota is a complex and diverse microbial community in the human gut which plays an important role in regulating human immune homeostasis, maintaining host mental health, and regulating brain function, behavior, and metabolism (7). The gut microbiota, enteric nervous system, autonomic nervous system, metabolic system, and central nervous system form a complex signalling network, constituting the gut microbiota-gut-brain axis (8). At the same time, the gut microbiota imbalance is closely related to tumorigenesis and progression, and some studies have found bidirectional regulation between adverse reactions induced by BC chemotherapy and the gut microbiota (9–11). In addition, recent evidence on the link between the gut microbiome and the brain reveals a new approach to CRF research. In fact, alterations in the gut microbiota are closely related to the pathogenesis of various neuropsychological diseases, such as chronic fatigue syndrome, depression, cognitive impairment, and CRF (2, 12–14).

Increasing evidence has shown that when the gut microbiota is imbalanced, gut microbes can destroy the permeability of the gut barrier, cause gut inflammation, peripheral blood inflammatory response, and then destroy the blood–brain barrier (BBB) function, leading to a central inflammatory response, inflammation, and dysfunction of the HPA axis, ultimately resulting in neurological dysfunction (15, 16). The gut barrier is an important part of the gut immune system and plays a crucial role in maintaining the balance between the host and gut microbiota. A large number of studies have confirmed that anti-cancer chemotherapy treatment can disrupt the gut barrier function, because it may damage the gut epithelial tissue and expose more intestinal contents to the crypts (17). At the same time, related studies have found that tight junction proteins ZO-1, occludin, claudin5, and the level of gut inflammation are important indicators for judging gut barrier function (18). In addition, a series of studies have shown that neuroinflammation is closely related to the occurrence and development of CRF. Gut microbiota imbalance, caused by chemotherapy treatment, can disrupt the function of the BBB, leading to an increase in central pro-inflammatory factors such as IL-1β, IL-6, and TNF-α, which can, in turn, cause the expression of HPA axis-related factors such as cortisol (CORT), corticotropin-releasing hormone (CRH), and disturb the adrenocorticotropic hormone (ACTH), exacerbating fatigue symptoms (19, 20).

Metabolomics is an emerging field and an important component in biomedical research which allows us to study the content of biological matrices at the molecular level. This method is particularly suitable for the discovery of biomarkers that reflect the homeostasis of the body during metabolic processes and dynamic responses to physiological stimuli. Related studies have found that fatigue is affected by the levels of certain amino acids in plasma and can alter the plasma metabolic profile (21). Meanwhile, other studies have confirmed that 40% of metabolites in the human body are closely related to the microbiota. The microbiota is a rich source of human metabolites and an important regulator of metabolite signal transduction (15, 22). Importantly, related studies have shown significant changes in serum metabolites in CRF patients compared with non-fatigue patients (23).

Acupuncture is a non-drug treatment method that involves inserting fine needles into specific acupoints in the human body and is also an adjunct and alternative therapy widely used in clinical practice (24). The National Institutes of Health (USA) has recognized acupuncture as an effective treatment for a variety of diseases. In addition, the World Health Organization (WHO) recommended the use of acupuncture for at least 100 diseases in 2003 (25). Due to the advantages of acupuncture in regulating various biological processes and pathways, it is widely used to treat adverse reactions caused by tumor therapy, such as fatigue, bone marrow suppression, vomiting, and sleep disorders (26). Importantly, acupuncture for breast CRF and CRF after breast cancer chemotherapy is effective (27). In addition, related studies have confirmed that acupuncture can regulate the disturbance of the gut microbiota and restore homeostasis by interfering with all aspects of the gut-brain axis (28).

Here, we investigated the alterations of acupuncture on fatigue-like behavior, gut microbiota, gut inflammation, neuroinflammation response, gut barriers, and the HPA axis in a CRF mouse model after breast cancer chemotherapy, as well as changes in serum metabolites. In addition, to clarify the role of the gut microbiota in gut-brain bidirectional communication, we performed gut microbiota functional prediction and correlation analysis between differential microbiota, serum differential metabolites, and other indicators.

## Materials and methods

### Animals and cells

SPF BALB/c female mice, 6-8 weeks old, body weight 15-18 g, provided by Beijing Weitong Lihua Laboratory Animal Science and Technology Co were used in this study. Animal welfare and experimental protocols were strictly in accordance with the Guide for the Care and Use of Laboratory Animals as well as the ethics and regulations of the Ethical Review Committee of Laboratory Animal Welfare of the First Affiliated Hospital of Henan University of Chinese Medicine (YFYDW2021012). The animals were raised in the SPF animal laboratory of the First Affiliated Hospital of Henan University of Chinese Medicine, with controlled room temperature at 20°C-25 °C, relative humidity at 40%-70%, and a 12 h day and night alternation. All mice were fed standard rodent chow and purified water and were adaptively fed for 1 week.

The Oncology Laboratory of Guang’anmen Hospital, Chinese Academy of Chinese Medical Sciences obtained 4T1-Luciferase mouse breast cancer cells from the National Cancer Institute (USA). Cells were routinely cultured in RPMI-1640 medium, placed in a 37°C, 5% CO2 incubator for culture, passaged once every 2-3 days, and used for experiments in the logarithmic growth phase.

### Grouping and modelling

**Figure 1:**
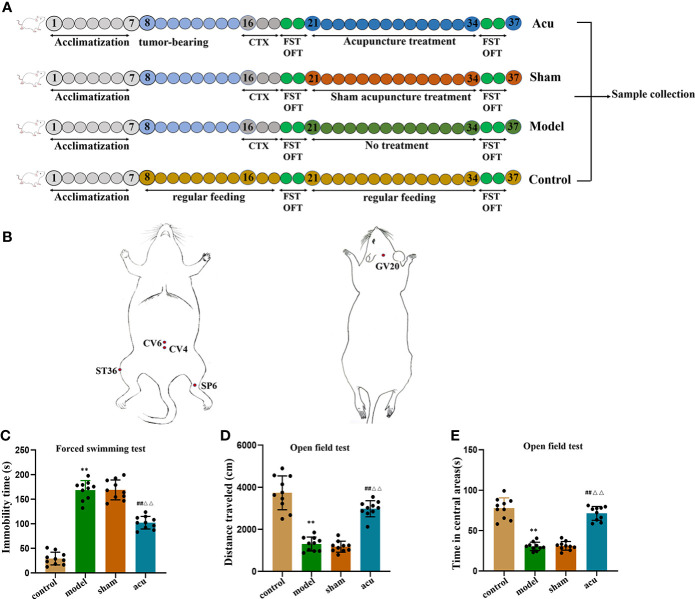
Acupuncture treatment exerts anti-fatigue effect in CRF mice after chemotherapy for BC. **(A)**, Experimental design. After acclimatization for 1 week, mice received tumor bearing for 7days, followed by intraperitoneal injection of CTX for three consecutive days, after behavioral testing, they were divided into acu group, sham group, and model group. The mice in the control group were given regular feeding throughout the whole process. **(B)** The localization of acupoints selected for acupuncture. **(C)** After tumor-bearing plus chemotherapy and acupuncture treatment, the forced swimming immobility time were measured in the FST. **(D)** After tumor-bearing plus chemotherapy and acupuncture treatment, the distance traveled were measured in the OFT. **(E)** After tumor-bearing plus chemotherapy and acupuncture treatment, the time spent in the central area was measured in the OFT. *n* = 10 mice in each group. ^**^ *P* < 0.01 versus control group; ^##^ *P* < 0.01 versus model group; ^△△^ *P* < 0.01 versus sham group.

Using the random number table method, forty female mice were randomly divided into 4 groups (n = 10/group): (1) the control group, in which the mice were fed normally without any intervention; (2) the model group, in which the mice were treated with subcutaneous tumor grafting and cyclophosphamide chemotherapy treatment; (3) the acupuncture (acu) group, in which the mice all received acupuncture treatment at corresponding treatment points after successful modelling; and (4) the sham acupuncture (sham) group, in which the mice were stimulated at non-acupuncture points near the corresponding treatment points (**Figure 1A**).

When mice were adaptively fed for a week, the concentration of 4T1 cells was adjusted to 2 × 10^6^/mL, and 0.1 mL of cells was subcutaneously inoculated into the fourth breast pad area of each mouse. When the tumor volume was approximately 0.5 cm^3^, the model group was given an intraperitoneal injection of cyclophosphamide (CTX) (Anderson ENDOXAN company, batch number: H20160467) 100 mg/(kg·d) for 3 consecutive days. Weight loss, loss of appetite, lethargy, slowness of movement, prolonged immobility time in forced swimming, and reduction of horizontal and vertical movement scores in the open field of mice in the model group were the criteria for successful modelling.

### Acupuncture treatment

The intervention started on the day after successful modelling and was performed for a total of 14 days. Acupoints of Zusanli (ST36), Sanyinjiao (SP6), Guanyuan (CV4), Qihai (CV6), and Baihui (GV20) were selected for acupuncture treatment based on acupuncture theory (29). The acupoint ST36 is located under the knee joint of the mouse and 0.3 cm below the fibular head in the muscle groove, and the SP6 is located 0.5 cm above the tip of the inner malleolus of the hind limb, behind the umbilicus. Moreover, CV4 is located 1 cm behind the umbilicus, CV6 is located 0.5 cm behind the umbilicus, and GV20 is located in the middle of the parietal bone (**Figure 1B**). After disinfection at the acupoints, sterile stainless steel disposable acupuncture needles (Hwato brand, length 13 mm, diameter 0.2 mm, Suzhou Medical Products Co., Ltd., Jiangsu Province, China) were inserted straight into the acupuncture points (ST36 and ST6) at depths of 3 mm and 1.5 mm. For acupoints CV4 and CV6, acupuncture needles were obliquely inserted to a depth of 1.5 mm. Acupoint GV20 received a needle inserted in parallel to a depth of 1 mm. The acupuncture needles were rotated left and right at a frequency of 60 times per minute for 20 seconds, operated once every 6 minutes, and pulled out after 30 minutes. The acupuncture course was 14 days of continuous treatment. In the sham group, stimulation was performed on the handles of the acupuncture needles at non-acupuncture points near the relevant treatment points in the mice. The stimulation time was the same as the acupuncture treatment time.

### Forced swimming test

Mice were placed alone in a transparent cylindrical bucket (25 cm height, 10 cm internal diameter) with 10-15 cm clean fresh water inside (23°C-25°C, 10-15 cm depth) and were placed in a dark environment on the second day after modelling and 14 days after intervention. After the mice were forced to swim undisturbed, researchers recorded them on video for 6 minutes. The immobility of mice in the last four minutes was counted to evaluate the physical strength and fatigue of mice. After each mouse was tested, the cylinder was refilled with clean fresh water.

### Open field test

An open square box with a size of 50 cm × 50 cm × 50 cm was used in this study and the bottom of the box was evenly divided into small cells of 5 × 5 with lines. The experiment was conducted in a quiet and dark environment, and the camera for recording was installed above the device. The device was cleaned with 75% alcohol after each mouse was tested. After the mice were placed in the center of the bottom of the box, the total distance travelled and time spent in the central area were analyzed by a motion tracking system.

### Sample collection and preparation

After the experiment, the mice were sacrificed with 1% pentobarbital sodium (50 mg/kg). The blood of the mice was collected by the orbital blood test and then the serum samples were extracted. Subsequently, the abdominal cavity was opened and part of the colon was cut out. After washing with PBS, the gut contents were aseptically collected and the gut tissue was also preserved. At the same time, the brain tissue was dissected and the hippocampus tissue was removed for preservation. All serum samples and tissue samples were stored in a refrigerator at -80°C.

### Western blot

Western blotting was performed to determine the protein levels of ZO-1, claudin-5, and occludin in tight junction proteins of gut tissue and the protein expression levels of the proinflammatory factors TNF-α, IL-1β, and IL-6 in the gut and hippocampus. Gut and hippocampal tissue samples were collected, lysis buffer was added, and the samples were centrifuged at 15000 × g at 4°C. The protein supernatant was then extracted and the protein concentration was measured by the BCA method. Subsequently, gel preparation, sample loading, electrophoresis, and membrane transfer were performed; 5% nonfat milk powder was applied for blocking for 1 h and primary antibody was added and incubated overnight at 4°C. Then, TBST was used to wash the membrane two times for 5 min each and a secondary antibody was added for 1 h at room temperature. Finally, we incubated the membrane in ECL luminescent solution for 3-5 min, conducted dark room exposure, scanned the strip, calculated the grey value, and analyzed and data.

### ELISA

The ELISA detection kits for CRH, CORT, and ACTH were equilibrated at room temperature for 30 minutes and after the samples were dissolved at room temperature, standards with different gradient concentrations were prepared and standard dilutions were added. Then, 50 μL of biotin-conjugated antibody was added to each well and the plate was sealed, placed on a horizontal shaker, shaken slightly, mixed, and incubated at room temperature for 2.5 h. Subsequently, we discarded the supernatant, added the detection antibody, incubated it at room temperature for 1 h, discarded the supernatant again, incubated it in the dark at room temperature, added the stop solution, mixed it thoroughly, and detected the absorbance OD value at 450 nm with a microplate reader. Finally, we drew a standard curve and calculated the content of corresponding factors in each group of samples.

### 16S rRNA sequencing

The stool samples of each group were removed from the -80°C refrigerator. Total genomic DNA from stool samples was extracted using the CTAB/SDS method and the concentration and purity were monitored by 1% agarose gels according to the concentration. The DNA was diluted to 1 ng/µL with sterile water. Primers were intended to amplify the region between the variable V3-V4 region of the 16S rRNA gene. PCRs were performed with 15 μL of Phusion^®^ High-Fidelity PCR Master Mix (New England Biolabs, USA), 0.2 μM forward and reverse primers, and approximately 10 ng of template DNA. The initial denaturation temperature was set at 98°C for 1 min, denaturation temperature was at 98°C for 10 s (30 cycles), annealing temperature was 50°C for 30 s, extension temperature was 72°C for 1 min, and final elongation temperature was 72°C for 8 min and stored at 10°C. PCR products were electrophoresed on a 2% agarose gel and purified using the Qiagen Gel Extraction Kit (Qiagen, Germany). A TruSeq DNA PCR-Free Sample Preparation Kit (Illumina, USA) was used to construct the library, which was assessed on an Agilent Bioanalyzer 2100 system. After the library was qualified, Illumina NovaSeq was used for 16S rRNA V4 sequencing. We spliced and filtered the original data to filter out contaminated data to obtain adequate accurate and reliable data.

Uparse software (Uparse v7.0.1001, http://drive5.com/uparse/) was used to cluster clean reads of all samples and sequence clustering was converted into operational taxonomic units (OTUs) by default with 97% identity. Species annotation analysis was performed on the Silva Database (http://www.arb-silva.de/), based on the Mothur algorithm, to study the difference and community composition of dominant species in different samples. Sample species diversity and abundance changes were analyzed through the Shannon and Simpson Alpha diversity-related indicators. Meanwhile, the metrics in our sample were calculated with QIIME (Version 1.7.0) and displayed with R software (Version 2.15.3). QIIME software (Version 1.9.1) was used to calculate beta diversity and principal coordinate analysis (PCoA) was mainly used to illustrate the similarities and differences in the tested samples. Finally, the differences in species composition and community structure between groups were further analyzed by LEfSe analysis and species with significant differences at each level were screened.

### Serum metabolomic analysis

#### Serum sample pretreatment

The serum was removed from the -80°C refrigerator, 200 μL of serum was added to 600 μL of cold methanol, the samples were vortexed for 1 min, and then sonicated twice at low temperature for 30 min each time, after which they were left to stand at 20°C for 1 h to precipitate proteins. The samples were then centrifuged at 14,000 × g for 20 min at 4°C, and the supernatant was transferred, vacuum freeze-dried, and stored in a refrigerator at 80°C until use. The samples were reconstituted with 100 μL of acetonitrile aqueous solution (1:1, V/V) and centrifuged at 4°C (14000 × g, 15 min), and the supernatants were collected for mass spectrometry analysis.

#### Chromatography and mass spectrometry conditions

Chromatographic separation of samples was carried out with an ultrahigh pressure liquid chromatograph (Agilent 1290 InfinityLC, USA), a chromatographic column (Waters, ACQUITY UPLC BEH Amide 1.7 μm, 2.1 mm×100 mm column, USA), column temperature: 25°C, injection volume: 2 μL, flow rate: 0.3 mL/min; mobile phase A: water+25 mmol/L ammonium acetate (Sigma, 70221, USA) + 25 mmol/L ammonia water; mobile phase B: pure acetonitrile. After the samples were separated by UHPLC, a Triple TOF 5600 + mass spectrometer (AB SCIEX, USA) was used for electrospray ionization (ESI) positive and negative ion mass spectrometry analysis. Electrospray ionization conditions: ion source gas 1:60, ion source gas 2:60, temperature: 600°C, voltage is ± 5500 V in positive and negative modes, mass scanning range is m/z: 60~1000 Da, first-level scanning range was 25~1000 Da, scanning time was 0.2 s, high-sensitivity mode was adopted. For the second mass spectrometry, the declustering voltage of the positive and negative modes was ±60 V and the high collision energy was (35 ± 15) eV.

#### Screening and identification of main differential metabolites and metabolic pathway enrichment analysis of differential metabolites

Peak area extraction, peak alignment, and retention time correction were performed using the XCMS program to remove ion peaks with group sums greater than 2/3. Then, Pearson correlation analysis and multivariate control (MCC) were performed on the QC samples, and Hotellings T2 analysis was performed on the overall sample. Subsequently, SIMCA-P 17 pattern recognition was used to perform multidimensional statistical analysis (unsupervised principal component analysis (PCA), supervised Orthogonal Projections to Latent Structures-Discriminant Analysis (OPLS-DA), and univariate statistical analysis (UVA)) to screen for differential metabolites. At the same time, KEGG annotation was performed on the differential metabolites, metabolite databases such as KEGG and PubChem were further mapped by the differential metabolites, and the metabolic pathways of the differential metabolites were enriched and analyzed.

### Statistics

The measured data are expressed as the mean ± standard deviation (±*s*). For comparison between groups, if the normality test is met, two independent samples *t* test is used; if the normality test is not met, Wilcoxon Man-Whitney U rank sum test for two-sample comparison is used. *P* < 0.05 was considered statistically significant. The significant differences between the groups were first analyzed by one-way analysis of variance (ANOVA) and when there was a significant interaction, Tukey’s HSD *post hoc* test was used for the next step of analysis for multiple comparisons. The Mann-Whitney U test and LDA effect size analysis (LEfSE) were used to analyze the composition and structural changes of the gut microbiota. The Spearman rank correlation analysis method was used to analyze the correlations between the gut microbiota and other indicators. Correlation graphs and heatmaps were drawn using GraphPad Prism 8 Software (GraphPad Software, Inc., San Diego, CA, USA) and open-source Cytoscape software (v.3.7.1, NIGMS, USA).

## Results

### Acupuncture treatment exerts an anti-fatigue effect in CRF mice after chemotherapy for BC

We first used FST and OFT experiments to explore the fatigue-like behavior of mice in each group after tumor-bearing plus chemotherapy and after acupuncture intervention. Compared with the control group, the forced swimming immobility time in the tumor-bearing plus chemotherapy group (model group) increased in the FST (**Figure 1C**). In the OFT, both the total distance travelled and the time spent in the central area decreased in the model group (**Figures 1D, E**). Then, we determined whether acupuncture intervention had an anti-fatigue effect. Compared to the model group, the immobility time of forced swimming in the acupuncture group (acu group) significantly decreased in the FST (**Figure 1C**), and the total distance travelled and the time spent in the central area in the OFL significantly increased (**Figures 1D, E**), while there was no difference between the sham acupuncture group (sham group) and the model group in the FST and OFT (**Figures 1C–E**). Meanwhile, compared with the sham group, the forced swimming immobility time of the acu group was also significantly shortened in the FST and the total distance travelled and the time spent in the central area in the OFT were significantly elevated in the OFT (**Figures 1C–E**). These data suggested that acupuncture treatment had anti-fatigue effects in CRF mice after chemotherapy for BC.

### Acupuncture treatment modulates the species abundance of the gut microbiota of CRF mice after chemotherapy for BC

**Figure 2:**
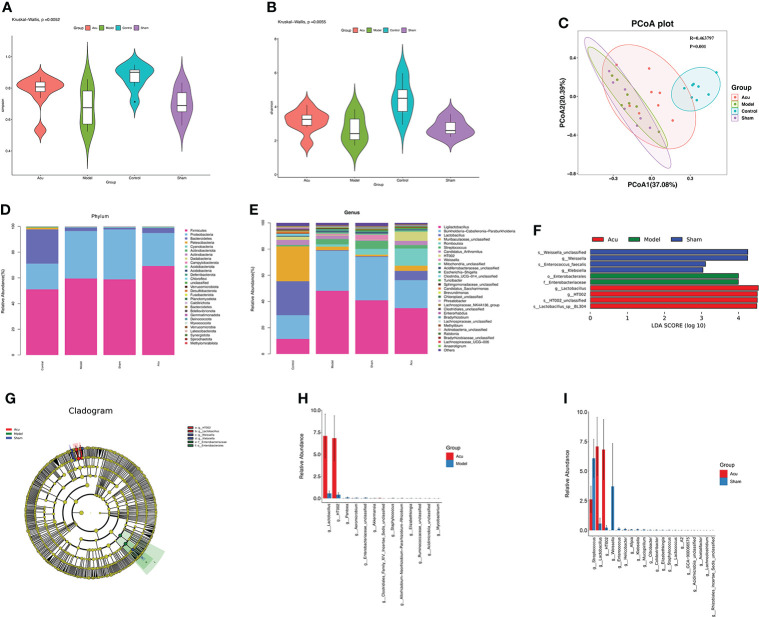
Acupuncture treatment modulates species’ abundance of gut microbiota of CRF mice after chemotherapy for BC. **(A, B)** The Shannon index (*P*=0.0052) and the Simpson index (*P*=0.0055) of acupuncture treatment on species richness and diversity were significantly different. **(C)** The PCoA data showed that the β-diversity between the acu group and the control group was more similar than that between the model group, the sham group and the control group (*P*=0.001, R=0.463797). **(D)** At the phylum level, phyla abundance changes in each group. **(E)** At the phylum level, the genus abundance changes in each group. **(F, G)** LEfSe analysis showed the community composition and species abundance in the acu, model and sham group. **(H, I)** The histogram shows the relative abundance of the different genera between the acu group and the model group and between the acu group and the sham group at the genus level.

Alterations in the gut microbiota are closely related to CRF. Therefore, we detected changes in the gut microbiota in CRF mice after chemotherapy for BC using 16S rRNA sequencing. Alpha diversity is a measure of species diversity and richness that calculates the Shannon and Simpson indices. The results showed that the Shannon and Simpson indices were significantly higher in the control group than in the model and sham groups, while the Shannon and Simpson indices of the acu group were significantly increased when compared with those of the model and sham groups (**Figures 2A, B**). Beta diversity analysis was performed using the Bray–Curtis distance-based principal coordinates analysis (PCoA) to detect the similarity of gut microbial community structure among samples. The PCoA results showed that the sample points of the model and sham groups were completely separated from the control group, while some of the sample points of the acu group were relatively close to those of the control group (**Figure 2C**). The above results showed that the flora abundance, diversity, and species differences of the gut microbiota of CRF mice after chemotherapy for BC had changed significantly and acupuncture treatment could effectively reverse this change.

According to the results of the relative abundance table of species, we counted the relative abundances of the top thirty species in each group at the phylum and genus levels and then drew abundance histograms. The composition of the gut microbiota composition in each group at the phylum level is shown in **Figure 2D**. Compared with the control group, the abundance of *Proteobacteria* was significantly increased and the abundance of *Bacteroidetes* and *Patescibacteria* were significantly decreased in the model and sham groups. While the abundance of *Firmicutes*, *Bacteroidetes*, and *Patescibacteria* in the acu group were significantly increased, the abundance of *Proteobacteria* was significantly decreased compared with the model and sham acupuncture groups. At the genus level, the abundance of *Burkholderia-Caballeronia-Paraburkholderia*, *Escherichia-Shigella*, and *Streptococcus* were higher, while those of *Lactobacillus*, *Muribaculaceae_unclassified*, *Candidatus_Arthromitus*, and *Clostridia_UCG-014_unclassified* were lower in the model group than in the control group. Compared to the model and sham groups, the abundance of *Burkholderia-Caballeronia-Paraburkholderia*, *Escherichia-Shigella*, and *Streptococcus* decreased, while those of *Lactobacillus*, *Muribaculaceae_unclassified*, *Candidatus_Arthromitus*, and *Clostridia_UCG-014_unclassified* increased in the acu group (**Figure 2E**). Linear discriminant analysis (LDA) effect size (LEfSe) revealed significant differences in species abundance among the groups. According to the LEfSe analysis and LDA score (LDA score ≥3), the abundance of *Lactobacillus* was higher in the acu group than in the model and sham groups (**Figures 2F, G**). Additionally, according to the species abundance table, significant difference analysis was performed between genus level groups. We found that the level of *Lactobacillus* significantly increased after acupuncture treatment (**Figures 2H, I**). All of these findings suggest that acupuncture treatment could improve the imbalance of gut microbiota and increase the content of beneficial bacteria in CRF mice after chemotherapy for BC.

### Acupuncture treatment ameliorates gut barrier and gut inflammation and neuroinflammation in CRF mice after chemotherapy for BC

**Figure 3:**
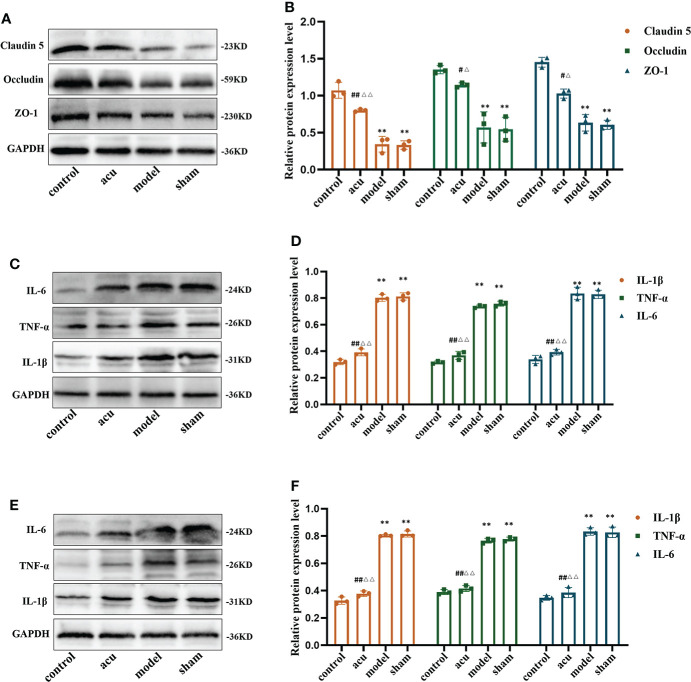
Acupuncture treatment ameliorates gut barrier, gut inflammation and neuroinflammation in CRF mice after chemotherapy for BC. **(A, B)** The expression levels of tight junction proteins ZO-1, Occludin, and Claudin 5 in gut. **(C, D)** The expression levels of pro-inflammatory factors IL-1β, IL-6, and TNF-α in the gut. **(E, F)** The expression levels of pro-inflammatory factors IL-1β, IL-6, and TNF-α in the hippocampus. *n* = 3 mice in each group. **P < 0.01 versus control group; ^#^ *P* < 0.05 versus model group, ^##^ *P* < 0.01 versus model group; ^△^ *P* < 0.05 versus sham group, ^△△^ *P* < 0.01 versus sham group.

In view of the disruption of the gut barrier function and the increased gut inflammatory and neuroinflammatory responses in CRF mice after chemotherapy for BC, we measured the expression of tight junction proteins in the gut and proinflammatory factors in the gut and hippocampus. Tight junction proteins can regulate paracellular permeability, increase gut epithelial barrier function, and maintain tight junction structure and barrier function. Related studies have found that the expression levels of the tight junction proteins ZO-1, occludin, and claudin-5 tend to decrease when intestinal permeability increases. In our study, we found that the protein expression levels of ZO-1, occludin, and claudin-5 were significantly decreased in the model group. Compared with the model group, the protein expression levels of ZO-1, occludin, and claudin-5 in the acu group were significantly increased, while there was no significant change in the sham group. Also, compared with the sham group, the protein expression levels of ZO-1, occludin, and claudin-5 in the acu group also increased significantly (**Figures 3A, B**). Subsequently, we found that CRF significantly enhanced the protein expression levels of the proinflammatory factors IL-1β, IL-6, and TNF-α in the gut and hippocampus compared to the control group, and all the levels of the three proinflammatory factors were significantly inhibited by acupuncture treatment (**Figures 3C–F**). The results confirmed that acupuncture treatment could improve gut barrier function and reduce the gut and hippocampal inflammatory responses in CRF mice after chemotherapy for BC.

### Acupuncture treatment improves the dysfunction of the HPA axis in CRF mice after chemotherapy for BC

**Figure 4:**
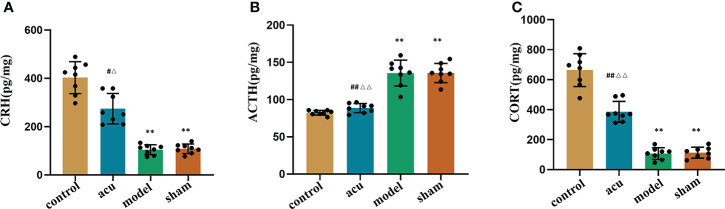
Acupuncture treatment improves the dysfunction of HPA axis in CRF mice after chemotherapy for BC. ELISA was used to detect the protein content changes of HPA axis-related factors CRH **(A)**, ACTH **(B)** and CORT **(C)** in the serum. *n* = 8 mice in each group. ^**^ *P* < 0.01 versus control group; ^#^ *P* < 0.05 versus model group, ^##^ *P* < 0.01 versus model group; ^△^ *P* < 0.05 versus sham group, ^△△^ *P* < 0.01 versus sham group.

The HPA axis includes cortisol (CORT), the corticotropin-releasing hormone (CRH), and the adrenocorticotropic hormone (ACTH). Numerous studies have shown that changes in the HPA axis play an important role in the development of fatigue. We used ELISA to detect the protein content changes of the HPA axis-related factors in this study. The research showed that CRF significantly reduced the levels of CRH and CORT and enhanced the levels of ACTH in serum compared to the control group. The expression levels of CRH and CORT were increased and the expression of ACTH was inhibited by acupuncture treatment compared to the model group. However, there were no significant changes in the levels of CRH, CORT, and ACTH in the sham and model groups (**Figures 4A–C**). Our findings implied that acupuncture treatment exerted an anti-fatigue effect by improving dysfunction of the HPA axis.

### Acupuncture treatment modulates serum metabolites and metabolic pathway changes in CRF mice after chemotherapy for BC

**Figure 5:**
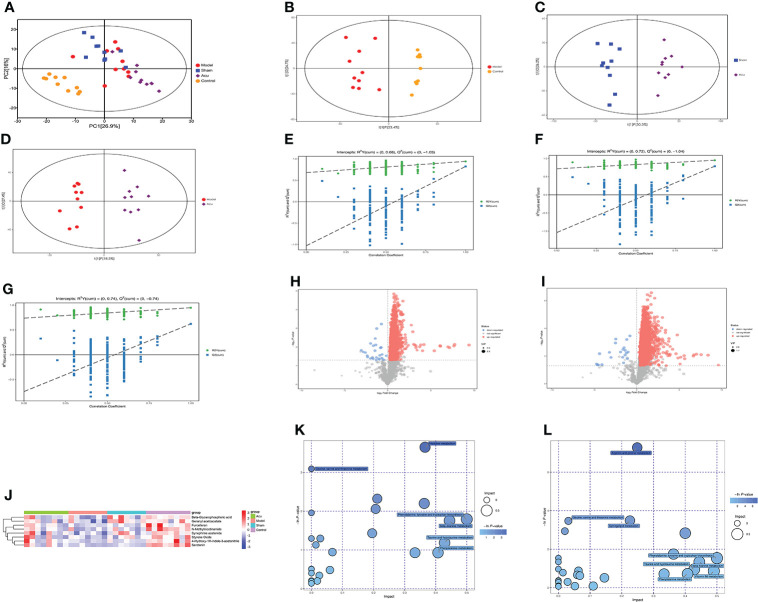
Acupuncture treatment modulates serum metabolites and metabolic pathways changes of CRF mice after chemotherapy for BC. **(A)** PCA was used to detect trends and outliers in the serum metabolome between groups. **(B)** Scores plots of OPLS-DA between the model and control groups. **(C)** Scores plots of OPLS-DA between the sham and acu groups. **(D)** Score plots of OPLS-DA between the model and acu groups. **(E–G)** The corresponding coefficient of loading plots between the model and control groups, sham and acu groups, and model and acu groups. **(H, I)** Volcano plot of the acu and model groups and acu and sham groups, where each point represents a metabolite. Red dots represent up-regulated metabolites, blue dots represent down-regulated metabolites. **(J)** Main differential metabolites between groups screened by VIP value and P value. **(K)** The related pathways screened out by comparing the model group and the control group. **(L)** The related pathways screened out by comparing the acu group and the model group.

Untargeted metabolomics analysis based on LC–MS was applied to investigate serum metabolite fluctuations in each group. Principal component analysis (PCA) was used to detect trends and outliers in the serum metabolome and the results suggested that the PCA distribution did not show any significant trends or differences between groups (**Figure 5A**). Therefore, to determine the real differences between the groups before and after acupuncture treatment, and to screen for effective differential metabolites, we used the statistical method of orthogonal projections to latent structures-discriminant analysis (OPLS-DA) to analyze the results of each group. As shown in the OPLS-DA score plots, the metabolites in the model group were significantly different from those in the control group (**Figure 5B**), and the metabolites in the acu group were also significantly different from those in the model and sham groups (**Figures 5C, D**). At the same time, there was good model fit and predictability between groups (**Figures 5E–G**). In addition, to describe the changes in metabolites between groups, volcano plots were analyzed. In comparison of the acu and model groups, there were more metabolites that were significantly upregulated than those that were significantly downregulated (**Figure 5H**). At the same time, the acu group also showed significantly more upregulated metabolites than significantly downregulated metabolites than the sham group. (**Figure 5I**).

According to the variable importance of the projection (VIP) values >1.0 and *P* value <0.05 of the OPLS-DA model, the influence of the expression patterns of each metabolite on each group was measured and the biologically significant differential metabolites were mined. Then, we constructed heatmaps of the four groups, visualized the results of the main differential metabolites, and summarized the distribution of the metabolites with the largest differences between the groups. We screened 8 major differential metabolites, and the serum levels of furcelleran, N-methylnicotinamide, 4-hydroxy-1H-indole-3-acetonitrile, beta-glycerophosphoric acid, geranyl acetoacetate, styrene oxide, serotonin, and synephrine acetonide were higher in the acu group than in the model and sham groups. These major differential metabolites were also highly expressed in the control group (**Figure 5J**). Subsequently, we used the Kyoto Encyclopedia of Genes and Genomes (KEGG) pathway database to analyze differential metabolite-related metabolic pathways. In the comparison between the model and control groups, histidine metabolism, phenylalanine, tyrosine and tryptophan biosynthesis, taurine and hypotaurine metabolism, phenylalanine metabolism, and beta-alanine metabolism were the affected pathways (**Figure 5K**). In the comparison of the acu and model groups, phenylalanine, tyrosine and tryptophan biosynthesis, beta-alanine metabolism, vitamin B6 metabolism, phenylalanine metabolism, and taurine and hypotaurine metabolism were the affected pathways (**Figure 5L**). Therefore, phenylalanine, tyrosine and tryptophan biosynthesis, taurine and hypotaurine metabolism, phenylalanine metabolism, and beta-alanine metabolism were the main differential metabolic pathways after acupuncture treatment. Overall, acupuncture treatment could modulate serum metabolites and metabolic pathway changes in CRF mice after chemotherapy for BC.

### Correlation analysis of differential bacterial genera and gut barrier, gut inflammation, neuroinflammation, HPA axis function changes, and differential serum metabolites

**Figure 6:**
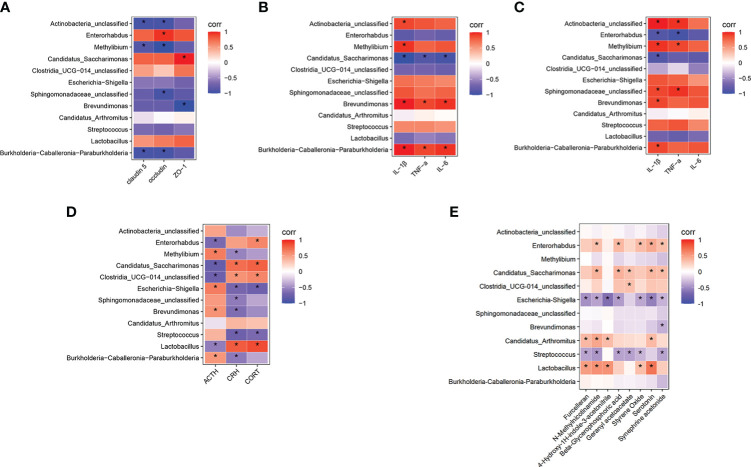
Correlation analysis of differential bacterial genera in model group and control group with gut barrier, gut inflammation, neuroinflammation, functional changes of HPA axis, and differential serum metabolites. **(A)** Correlation analysis between the differential bacterial genera and the gut tight junction protein. **(B)** Correlation analysis between the differential bacterial genera and the gut inflammation. **(C)** Correlation analysis between the differential bacterial genera and the neuroinflammation. **(D)** Correlation analysis between the differential bacterial genera and the HPA axis function changes. **(E)** Correlation analysis between the differential bacterial genera and the differential serum metabolites. The color of the grid represents the correlation analysis value of the Spearman correlation analysis. Red grids represent positive correlations and blue grids represent negative correlations. The color-coded scale represents the correlation analysis value of the heatmap, with darker red or blue indicating higher correlation values. ^*^ *P* < 0.05 for differential bacterial genera, gut barrier, gut inflammation, neuroinflammation, functional changes of HPA axis and differential serum metabolites.

Ultimately, we used the Spearman’s correlation analysis method to analyze the relationship between the model group and the control group of differential bacterial genera and gut barrier, gut inflammation, neuroinflammation, HPA axis function changes, and differential serum metabolites. In the resulting heatmaps comparing the model and control groups, the analysis revealed strong correlations between the differential gut microbiota and other indices. As shown in **Figures 6A, D**, *Burkholderia-Caballeronia-Paraburkholderia*, *Streptococcus*, and *Escherichia-Shigella* showed negative correlations with gut tight junction proteins, CORT and CRH, while *Lactobacillus* and *Clostridia-UCG-014-unclassified* showed positive correlations with gut tight junction proteins, CORT and CRH. *Streptococcus*, *Brevundimonas*, *Escherichia-Shigella*, and *Burkholderia-Caballeronia-Paraburkholderia* showed positive correlations with gut inflammation, neuroinflammation, and ACTH. However, *Lactobacillus* and *Clostridia-UCG-014-unclassified* showed negative correlations (**Figures 6B–D**). In addition, *Lactobacillus*, *Candidatus Arthromitus*, and *Clostridia-UCG-014-unclassified* showed positive correlations with the differential metabolites furcelleran, N-methylnicotinamide, 4-hydroxy-1H-indole-3-acetonitrile, beta-glycerophosphoric acid, geranyl acetoacetate, styrene oxide, serotonin, and synephrine acetonide while *Burkholderia-Caballeronia-Paraburkholderia*, *Streptococcus*, and *Escherichia-Shigella* showed negative correlations with the differential metabolites (**Figure 6E**).

## Discussion

Breast cancer is one of the most common malignant tumors in women worldwide. According to the latest global cancer data released in 2020 by the World Health Organization’s International Agency for Research on Cancer (IARC), the incidence of breast cancer ranks first in the world (3, 30). At the same time, with the deepening of breast cancer research and the advancement of various monitoring methods and treatment technologies, the survival time of breast cancer patients has been significantly prolonged (31, 32). However, most breast cancer survivors have adverse reactions, such as CRF, due to disease and treatment processes, and 60% of breast cancer patients will experience moderate to severe fatigue symptoms within 12 months after chemotherapy (4, 5, 33). Additionally, compared with general fatigue, CRF experienced by tumor patients is characterized by rapid development, long duration, and a severe degree that cannot be relieved after rest. Ultimately, it will negatively affect the patient’s physical, psychological, and social quality of life (34, 35). To explore methods and drugs for the treatment of CRF, many scholars in previous studies have prepared animal models of CRF induced by chemotherapy. For example, Zombeck used doxorubicin chemotherapy plus the treadmill to create a mouse model of fatigue (36). Some researchers established a tumor-related fatigue model by inoculating mice with breast cancer cells, assessed fatigue through OFT, TST, and FST experiments, administered paclitaxel chemotherapy, and found that chemotherapy can aggravate the fatigue of mice (37). In the present study, we used tumor bearing and chemotherapy to prepare a CRF mouse model of breast cancer after chemotherapy and used OFT and FST behavioral experiments to evaluate the degree of fatigue. Our results revealed that the FST immobility time was increased and both the total distance travelled and the time spent in the central area were decreased in the OFT in the CRF mouse model of BC after chemotherapy with severe fatigue.

Acupuncture has important clinical application value in the treatment of CRF. Especially in those patients undergoing chemotherapy and radiotherapy-induced fatigue, acupuncture treatment can significantly improve fatigue symptoms and improve overall quality of life. In addition, acupuncture at Zusanli (Stomach Meridian, ST36) has various effects such as anti-inflammatory, enhancing immune function, anti-fatigue, anti-oxidation, and promotion of the recovery of various wasting diseases. Sanyinjiao (Spleen Meridian, SP6) and Baihui (GV20) can relieve pain, anxiety, depression, fatigue, and other mood-related diseases. Qihai (CV6) and Guanyuan (CV4) can regulate the qi in the body, enhance the flow of qi, soothe the viscera, and then significantly relieve the symptoms of discomfort in the body, allowing the body to rest (38–40). A study confirmed that after acupuncture at Zusanli (ST36), Sanyinjiao (SP6), Qihai (CV6), and Guanyuan (CV4), the degree of fatigue of breast cancer patients in the convalescent stage was significantly lower than that of the sham acupuncture group, and the quality of life was significantly improved. Meanwhile, according to other studies, acupuncture treatment could improve the quality of life of CRF patients and have therapeutic potential in the management of CRF for cancer survivors (27, 41, 42). Therefore, in our study, we selected the four acupoints Zusanli (ST36), Sanyinjiao (SP6), Baihui (GV20), Qihai (CV6) and Guanyuan (CV4) to improve fatigue. More importantly, our results confirmed that acupuncture treatment could exert an anti-fatigue effect in CRF mice after chemotherapy for BC by reducing the forced swimming immobility time in the FST, increasing the total distance travelled, and the time spent in the central area in the OFT.

It is well known that gut microbes play an important role in the occurrence and development of central nervous system diseases and they are a key link between the gut and central nervous system (8, 43). Previous studies have confirmed that CRF can cause changes in the gut microbiome and gut microbiome imbalance is also involved in the occurrence and development of CRF (12–14). In addition, acupuncture treatment can improve the abundance of the gut microbiome and the changes in bacterial species regarding depression, fatigue, cognitive impairment, sleep disturbance and other emotional diseases related to the central nervous system (27, 44, 45).

In a large number of CRF-related microbiota studies, *Proteobacteria*, which are gram-negative bacteria, are involved in destroying the intestinal epithelial function, inhibiting the proliferation of beneficial bacteria, destroying the structure of flora, interfering with normal anti-inflammatory and immunosuppressive mechanisms, disrupting tumor immune surveillance, inducing adverse tumor treatment reactions, and promoting the occurrence of CRF (46, 47). Other related data confirmed that *Bacteroidetes* and *Firmicutes* can promote the metabolism of ginsenosides, improve the inflammatory response, and indirectly improve the fatigue state (50, 48). Moreover, a study found that through Chinese medicine treatment, the abundance of *Bacteroidete*s and *Firmicutes* was significantly increased while that of *Proteobacteria* was decreased at the phylum level in colon cancer CRF mice (49). This is consistent with our findings that at the phylum level, that the abundance of *Bacteroidete*s was significantly decreased and that of *Proteobacteria* was significantly increased in CRF mice after breast cancer chemotherapy, whereas the abundance of *Bacteroidete*s and *Firmicutes* was elevated and that of *Proteobacteria* was reduced after acupuncture treatment. Chemotherapy may disrupt the homeostasis of the gut microbiome, resulting in significant decreases in probiotics such as *Bifidobacteria* and *Lactobacillus* and increases in opportunistic pathogens such as *Staphylococcus*, *Streptococcus*, and *Escherichia coli* (50). Further research has shown that *Lactobacillus* probiotics can prevent adverse reactions such as cardiotoxicity, intestinal toxicity, fatigue, and sleep disturbance caused by chemotherapy drugs (51). Zulpa found that *Burkholderia* may induce inflammatory reactions and cause a series of adverse reactions in cancer patients after chemotherapy (52). Some studies found correlations between depression recovery and the inhibition of *Clostridium*, *Candidatus Arthromitus*, and *Lactobacillus* by antibiotics and further confirmed that *Clostridium*, *Candidatus Arthromitus*, and *Lactobacillus* may inhibit the progression of depression (53). At the genus level, we found that the levels of *Lactobacillus*, *Candidatus Arthromitus*, and *Clostridia_UCG-014_unclassified* were decreased and those of *Burkholderia-Caballeronia-Paraburkholderia*, *Streptococcus*, and *Escherichia-Shigella* were increased in CRF mice after breast cancer chemotherapy. The abundance of *Lactobacillus*, *Candidatus Arthromitus*, *Clostridia_UCG-014 unclassified*, *Streptococcus*, *Escherichia-Shigella*, and *Burkholderia-Caballeronia-Paraburkholderia* were reversed after acupuncture treatment. Taken together, acupuncture treatment of CRF after breast cancer chemotherapy can effectively regulate gut microbiota imbalance, increase the abundance of beneficial bacteria, and reduce the abundance of pathogenic bacteria.

Related studies have demonstrated that gut microbiota imbalance can damage gut tight junction proteins, leading to increased gut permeability and gut inflammation, and can further damage to the blood brain barrier, causing central inflammation in central nervous system diseases (54, 55). A study confirmed that the gut barrier of breast cancer patients was severely damaged and the expression levels of the tight junction proteins ZO-1 and occludin were significantly decreased (56). Another interesting study confirmed that central inflammatory factors were highly expressed in patients with cognitive impairment caused by breast cancer chemotherapy (57). In the present study, we found that CRF after breast cancer chemotherapy disrupted tight junction proteins (ZO-1, occludin, and claudin-5) in the gut barrier and stimulated inflammatory cytokine responses (IL-1β, IL-6, and TNF-α) in the gut and hippocampus, which were significantly reversed by acupuncture treatment. Cancer and its treatment can lead to intestinal mucosal barrier dysfunction and increased intestinal permeability, which can lead to systemic and intestinal immune and inflammatory responses. There is also evidence that the gut microbiome plays an important role in the regulation of gut and systemic inflammation levels. Related studies have found that chemotherapy-induced increases in the abundance of pathogenic bacteria *Clostridium cluster XI* and *Enterobacteriaceae* are closely related to the severity of fatigue, intestinal barrier function, and inflammatory response (58). Another study has shown that gut microbiome dysbiosis can damage the integrity of the blood-brain barrier, cause central inflammatory responses, and lead to mood disorders in patients (59). According to the results of our correlation analysis, it was suggested that there are correlations between tight junction proteins, gut inflammation, hippocampal inflammation, and gut microbiota. These findings suggested that acupuncture may play an anti-fatigue effect by regulating gut microbiota and improving gut barrier function, gut inflammation, and central inflammation.

The HPA axis includes CORT, CRH, and ACTH. Numerous studies have shown that central inflammatory responses can disrupt the function of the HPA axis, reduce cortisol synthesis and release, and cause CRF (24, 60). A randomized controlled trial found that the serum ACTH level in the CRF group was significantly higher than that in the non-fatigue group, and ACTH was highly positively-correlated with CRF (61). Weinrib et al. found that the change in serum CRH in ovarian cancer patients was negatively correlated with CRF (62). Our findings showed that CRF, after breast cancer chemotherapy, significantly reduced the levels of CRH and CORT and enhanced the levels of ACTH in serum, while the levels of CRH, CORT, and ACTH were also restored by acupuncture treatment. At the same time, we also confirmed that the main differential microbiota in the gut were closely related to the expression of HPA axis-related factors by correlation analysis. This result confirmed that acupuncture can improve the dysfunction of HPA axis through the gut microbiota-gut-brain axis pathway, thereby improving fatigue symptoms.

It is now generally accepted that the gut microbiota may play an important role in the regulation of metabolites and metabolic pathways. In our research, major differential metabolites, such as N-methylnicotinamide, beta-glycerophosphoric acid, geranyl acetoacetate, and serotonin, and related metabolic pathways, such as phenylalanine, tyrosine and tryptophan biosynthesis and beta-alanine, taurine and hypotaurine metabolism, were found. N-Methylnicotinamide is the final product of nicotinamide metabolism, and nicotinamide is involved in the tryptophan nicotinic acid pathway. One study found that altered levels of N-methylnicotinamide disrupted serotonin biosynthesis in people with depression. Serotonin is the precursor of tryptophan, which is closely related to central psychiatric diseases (63). Some evidence suggests that ketone bodies related to butyric acid structure (such as acetoacetate) play an important role in neurological disorders and psychiatric diseases (64, 65). In this study, we found that serotonin, N-methylnicotinamide, and geranyl acetoacetate in the model group were significantly lower than those in the control group but higher than those in the model group after acupuncture treatment. Phenylalanine and tryptophan are essential amino acids, and tyrosine can be synthesized from phenylalanine. Relevant studies have found that they are closely related to depression, CRF, and other psychiatric diseases (66). Another study found that alterations in tryptophan biosynthesis contribute to the development of CRF (67). Taurine, one of the most abundant amino acids in the central nervous system, has been shown to protect the brain by inhibiting central nervous system inflammation and improving learning and memory (68). In addition, it can improve depressive symptoms by regulating HPA axis function (69). Some studies have found that alanine is not only a potential neurotransmitter of glycine receptors but also a component of sarcosine and an inhibitor of taurine transport, which may be related to the recovery of spatial memory in the brain (70, 71). Another study confirmed that changes in alanine metabolism are closely related to changes in hippocampal function and improvement of brain memory deficits (72, 73). Taken together with our findings from the differential metabolites and the metabolic pathway analysis, compared to the control group, CRF after breast cancer chemotherapy caused changes in differential metabolites and the metabolic pathways of phenylalanine, tyrosine and tryptophan biosynthesis, taurine and hypotaurine, and beta-alanine. However, these differential metabolites and metabolic pathway changes were reversed after acupuncture treatment. Meanwhile, through the correlation analysis of differential metabolites and differential microbiota, we found that the changes in microbiota after acupuncture treatment can affect the changes in metabolites in serum. Therefore, our study confirmed that acupuncture treatment can affect the changes of serum metabolites and metabolic pathways by modulating the changes of gut microbiota in CRF model mice.

We acknowledge several limitations to this study. First, metabolic levels in gut contents were not explored in this study. Further studies with changes in gut microbial metabolites are needed to confirm the results. Second, more brain markers associated with fatigue have not been studied, so more research is needed to validate. Third, further validation should be carried out based on the screened differential bacteria to make this study more complete.

## Conclusion

In conclusion, our findings revealed that acupuncture treatment can modulate the gut microecological balance in a CRF mouse model after breast cancer chemotherapy and improve the changes in serum metabolites by regulating the gut microbiota while ameliorating gut barrier function, inhibiting gut inflammation and the neuroinflammatory response, and improving HPA axis dysfunction, thereby relieving symptoms of fatigue. All of these results confirmed that the anti-fatigue mechanism of acupuncture treatment may be closely related to the gut microbiota-gut-brain axis. In addition, this study provides a new reference for basic and clinical research on CRF after breast cancer chemotherapy.

## Data availability statement

The datasets presented in this study can be found in online repositories. The names of the repository/repositories and accession number(s) can be found below:https://www.jianguoyun.com/p/DalQoV0Q17_hBRibpbkEIAA.

## Ethics statement

This study was reviewed and approved by Ethical Review Committee of Laboratory Animal Welfare of the First Affiliated Hospital of Henan University of Chinese Medicine.

## Author contributions

ZL, XF contributed to the conception of the study. ZL, RL reviewed the literature and drafted this review. KS, YG contributed to the data curation and formal analysis of the study. ZL, LF, YF, XR performed the experiment, investigation and methodology. KS, JG contributed significantly to software analysis, gave critical comments and revised the manuscript. JG, XF were committed to the supervision and visualization of the manuscript. All authors contributed to the article and approved the submitted version.

## Funding

This research was supported by the National Natural Science Foundation of China Joint Fund (U2004131), Henan Province Chinese Medicine Scientific Research Special (2022ZY1019).

## Acknowledgments

We especially wish to thank the First Affiliated Hospital of Henan University of Chinese Medicine and SHANGHAI BIOTREE BIOMEDICAL TECHNOLOGY CO., LTD for providing laboratory equipment.

## Conflict of interest

The authors declare that the research was conducted in the absence of any commercial or financial relationships that could be construed as a potential conflict of interest.

## Publisher’s note

All claims expressed in this article are solely those of the authors and do not necessarily represent those of their affiliated organizations, or those of the publisher, the editors and the reviewers. Any product that may be evaluated in this article, or claim that may be made by its manufacturer, is not guaranteed or endorsed by the publisher.
